# Local concepts of anemia-related illnesses and public health implications in the Taabo health demographic surveillance system, Côte d’Ivoire

**DOI:** 10.1186/2052-1839-13-5

**Published:** 2013-05-06

**Authors:** M’Bra KD Kouadio, Aurélie A Righetti, Noël N Abé, Rita Wegmüller, Mitchell G Weiss, Eliézer K N’Goran, Jürg Utzinger

**Affiliations:** 1 Département d’Anthropologie et de Sociologie, Université Alassane Ouattara, Bouaké, Côte d’Ivoire; 2 Department of Epidemiology and Public Health, Swiss Tropical and Public Health Institute, Basel, Switzerland; 3 University of Basel, Basel, Switzerland; 4 Laboratory of Human Nutrition, Institute of Food, Nutrition, and Health, ETH Zurich, Zurich, Switzerland; 5 Unité de Formation et de Recherche Biosciences, Université Félix Houphouët-Boigny, Abidjan, Côte d’Ivoire; 6 Centre Suisse de Recherches Scientifiques en Côte d’Ivoire, Abidjan, Côte d’Ivoire

**Keywords:** Anemia, Behavior, Blood, Côte d’Ivoire, Cross-sectional study, Cultural concepts, Etiology, Knowledge, Mixed methods approach, Public health

## Abstract

**Background:**

A 14-month prospective longitudinal study conducted in the Taabo health demographic surveillance system (HDSS), south-central Côte d’Ivoire, revealed high prevalence of anemia in different population groups in three types of settings (i.e., small town, village, and hamlet). Demographic parameters and several variables related to parasitic infections, micronutrient status, and inflammation were significantly associated with higher odds of anemia. However, cultural concepts and knowledge of various anemia-related illnesses and their relation with people’s behaviors have not been investigated.

**Methods:**

Sixteen focus group discussions and six key informant interviews were performed with village authorities, health workers, and traditional healers. Questionnaires were administrated to 200 school-aged children and 115 young women. Of these individuals, 206 participated in the preceding longitudinal study, whereas the remaining 109 people were not exposed to prior research, but had similar age and sex profiles. Mean prominence of participants’ responses was compared between groups of participants and across study settings.

**Results:**

Local concepts of anemia-related illnesses referred to its perceived causes based on two logical frameworks – biomedical and sociocultural – although a clear distinction was often blurred. We found few differences in knowledge, beliefs, and behaviors across study settings and between participants who were exposed to prior research and newly recruited ones. Malaria und nutritional issues as understood and managed by the population differed from definitions and recommendations provided by the health system. Malaria was not acknowledged as an exclusive mosquito-transmitted disease and participants referred to the quantity, rather than the quality, of food when talking about nutritional issues.

**Conclusions:**

Local concepts and ideas about anemia have public health implications, inasmuch as they are related to people’s attitudes, risk-related and help-seeking behaviors, which in turn might affect their health status. Local terminology and beliefs about anemia and malaria should be carefully considered when developing health intervention and education programs. The similarity in knowledge about anemia-related illnesses and associated behaviors, regardless of study setting and prior exposure to research, suggests that a uniform communication strategy may be used to develop education programs and awareness campaigns aimed at the prevention and control of anemia in south-central Côte d’Ivoire.

## Background

Anemia, a term referring to a reduction in the number of red blood cells (RBC), hemoglobin (Hb) concentration, or oxygen-binding capacity of Hb, affects all population groups. Indeed, an estimated 2 billion people suffer from this condition worldwide [1]. Pregnant women and young children and, more generally, the poorest of the poor who live in settings where malnutrition and infectious diseases are widespread, are most affected [1,2]. In the humid tropics, anemia is multifactorial with malaria, iron deficiency, and helminth infections among the most important contributing factors to low Hb levels [3-5].

A situation analysis is the first step in developing education and intervention programs. Such an analysis not only entails defining the epidemiology or the extent of a given condition, but also requires assessing the health and economic status of residents, and studying people’s perceptions and attitudes toward the disease and potential interventions. Knowledge and related behaviors might vary depending on many parameters (e.g., demographic, sociologic, and economic parameters, genders, exposure to prior research or programs, season, etc.) [6-8].

In a preceding 14-month prospective, longitudinal study carried out in the Taabo health demographic surveillance system (HDSS) in south-central Côte d’Ivoire, which commenced in April 2010, we investigated the association between biomedical variables and anemia among infants (6–23 months), school-aged children (6–8 years), and young women (15–25 years) from three different settings. We found that, depending on age groups, *Plasmodium falciparum* and *Schistosoma haematobium* infections, inflammation, cellular iron deficiency, and chronic malnutrition were significantly and positively linked to the prevalence of anemia in this area [9,10].

Previous questionnaire-based studies pursued in sub-Saharan Africa primarily focused on knowledge, attitudes, practices, and beliefs (KAPB) among specific population groups, emphasizing a single etiological agent of anemia, such as malaria [11-13], soil-transmitted helminth infectons [14], iron deficiency [15], or sickle-cell trait [16]. Some studies have investigated local concepts related to malaria [13,17]. An ethnographic study carried out in Abidjan highlighted the implications of community understandings in the prevention and control of malaria [18]. However, basic concepts of blood and various anemia-related illnesses, the perception of the multifactorial etiology of anemia and their public health implications have yet to be characterized.

The purpose of this study was to deepen our knowledge of local concepts of blood and anemia in three settings of the Taabo HDSS. The specific objectives were (i) to define local concepts related to blood and anemia; (ii) to investigate the relationship between these concepts and local health problems; and (iii) to assess the heterogeneity of this relationship throughout the study area. Potential implications for public health are discussed placing emphasis on how findings affect risk-related and help-seeking behaviors.

## Methods

### Ethical considerations

The study protocol was approved by the institutional research commission of the Swiss Tropical and Public Health Institute (Swiss TPH; Basel, Switzerland, reference no. FK 96). Ethical approval was granted by the ethics committee of Basel (EKBB, reference no. 252/09) and Côte d’Ivoire (reference no. 1086 MSHP/CNER). Village chiefs, participants, and parents/guardians of children were informed about the purpose and procedures of the study. Written informed consent (or fingerprints of illiterate people and minors) was obtained from all interviewed participants and the parents/guardians of children <16 years of age.

### Study area and design

The study was conducted in the Taabo HDSS. Taabo Cité, the only small town within this HDSS is located some 160 km north-west of Abidjan, the economic capital of Côte d’Ivoire, and some 60 km south of Yamoussoukro, the political capital. Taabo HDSS is part of the Agnéby-Tiassa region, one of the 30 new administrative regions of Côte d’Ivoire designated in September 2011. The study area lies in the V-Baoulé, a transition zone from rainforest in the South to Savannah in the North, more precisely in the Eburean climatic area. There are four seasons: (i) a long rainy season lasting from April to July; (ii) a short dry season in the months of August and September; (iii) a short rainy season in October and November; and (iv) a long dry season, lasting from December to March. There are many small valleys in an overall flat landscape. The ferralitic soils are favorable to many types of cultivations found in forest zones (e.g., cacao, cassava, coffee, and rubber) and in Savannah areas (e.g., cassava and yam). The large hydroelectric dam, built in the late 1970s on the Bandama River, is a central feature of the area [19].

The design of our study was a cross-sectional survey, using a mixed methods approach (qualitative and quantitative), done in February 2012. The study was integrated into a larger project with the overarching goal to deepen our understanding of the etiology of anemia in the Taabo HDSS, and to investigate potential preventive and control measures [9]. Preceding the current work, from April 2010 to June 2011, we monitored anemia longitudinally in three age groups (infants, school-aged children, and women) in three settings of the Taabo HDSS (Ahondo, one of 14 villages; Katchénou, a former hamlet; and Taabo Cité). The three study cohorts were selected for their high risk of anemia or the high prevalence of parasitic infections, or both. The sample size of the three study cohorts was calculated based on the assumed prevalence of anemia in the Taabo area [9]. We found that several variables were significantly associated with anemia, including parasitic, nutritional, inflammatory, and demographic variables [10,20].

The current KAPB survey aimed at furthering our understanding of the local cultural knowledge and beliefs about anemia and their relation with people’s behaviors. Participants were selected according to the following criteria. First, we invited all school-aged children and women who had participated in one of the two last cross-sectional surveys of the previous longitudinal study (n=206). Additionally, we recruited 109 individuals who had not been exposed to prior research about anemia, but were otherwise comparable to the participants of the 14-month prospective longitudinal monitoring in terms of age, sex, and setting. Overall, the study was conducted in five localities that were representative of the three main types of settlement found in the Taabo area, namely (i) small town, (ii) village, and (iii) hamlet (i.e., small village that usually lacks social structures such as health facility and school, and is not officially registered as a village in the national registry). This sampling frame allowed us to minimize selection bias and, although not a main objective of this study, gave us the opportunity to investigate whether the longitudinal monitoring had an influence on people’s knowledge and beliefs about anemia.

Taken together, our study was conducted in (i) Taabo Cité, a small town with approximately 7,000 inhabitants, a district hospital with 12 beds staffed by a medical doctor and a surgeon, where both participants and non-participants to the longitudinal monitoring were enrolled; (ii) Ahondo, a village located in close proximity to Lake Taabo where all children (aged 6–8 years at baseline) and women (aged 15–25 years at baseline) were exposed to prior research, and where there is a health dispensary with a nurse; (iii) Sahoua, a neighboring village of Ahondo where people have not been exposed to the longitudinal study; (iv) Katchénou, a former hamlet enrolled in the longitudinal survey, where there was no health dispensaries at the time of the study; and (v) Amani Kouadiokro, a neighboring and very similar hamlet of Katchénou with no prior research. Depending on meteorological conditions, the population of both hamlets can access the health dispensary of Sokrogbo, located some 5 km away.

### Characteristics of target and main ethnic groups

Study respondents were school-aged children (8–10 years) and young women of reproductive age (17–27 years). According to the Taabo HDSS database, in February 2012, 40,574 people were registered as permanent residents in the Taabo HDSS.

The construction of a hydroelectric dam on the Bandama River in the late 1970s attracted people from different parts of Côte d’Ivoire and neighboring countries to Taabo, and hence the population has become quite cosmopolitan. Indigenous people are members of the Akan (mainly Baoulé) and the Krou ethnic groups (Bété), two of the four large groups of Côte d’Ivoire. Migrants from other regions of Côte d’Ivoire include people from the four ethnic groups, Akan (other Baoulé, Abidji, Agni, Akyé, Alladjan, etc.), Krou (Dida, etc.), Gur (Koulango, Senoufo, etc.), and South and North Mandé (South Mandé: Yacouba, Gouro, etc.; North Mandé: Malinké, Dioula, Kôyaka, etc.). Migrants from other nations mainly come from other African countries, especially from Burkina Faso, Mali, and Nigeria.

### Study instruments

Both qualitative and quantitative methods were used to gather information about anemia-related illnesses from school-aged children and young women, as well as health workers, traditional healers, and other village authorities. First, an information meeting was organized with all community chiefs from Taabo Cité in order to inform and mobilize the communities about the upcoming survey. During this meeting, local terms for blood and anemia were gathered. Second, questionnaires were pre-tested, and administered to women and school-aged children to assess KAPB about blood and various anemia-related illnesses for quantitative analysis. The questionnaires were divided in six parts: (i) sociodemographic parameters; (ii) local concepts and knowledge of blood and anemia-related illnesses; (iii) experience of anemia-related illnesses; (iv) prevention of anemia-related illnesses; (v) reported treatment-seeking behavior; and (vi) 24-hour food recall and nutritional habits. Results from our previous epidemiological study and literature review informed the development of our questionnaire and the categories for responses. The prominence of coded categories was based on whether responses identifying that category were reported spontaneously in response to an open question, only in response to probing for that category, or not reported at all. If a category was spontaneously reported, this gave a prominence of 2. If the category was only reported after probing, this gave a prominence of 1. A category not reported received prominence 0. This grading system allows calculating a mean prominence for each category. Multiple responses were permitted. The second author (AAR) coded spontaneously reported responses with reference to the categories of probed questions. The percentage of reporting in each category spontaneously and after having been probed is reported in the Tables.

Questionnaires were pre-tested in two steps. First, five questionnaires were administrated by the first author (MKDK) to check if children and women could understand the questions, to determine whether to add probed questions and to refine categories. For instance, the question “what would you do if you have anemia?” was not posed to children as the pretest showed that they were unable to respond without asking for the parent’s opinion.

Second, four field enumerators were trained to administer questionnaires. Each enumerator administered at least one questionnaire to a child and a woman in French and in Baoulé, under the supervision of two of us (MKDK and AAR). This training was continued until each enumerator was at ease with the questionnaire. Field workers were fluent in French, Baoulé, and Dioula, the three main local languages. In the rare cases that a participant did not understand any of these languages, a third person helped with translation.

Focus group discussions (FGDs) were conducted by the first author (MKDK) in French or Baoulé and tape recorded by the second author (AAR). In each locality, FGDs were conducted with children aged 8–10 years, women aged 17–27 years, village authorities and, in Taabo Cité, with the medical staff of Taabo General Hospital, to collect in-depth information about specific questions (Table 1). Groups included between 8 and 12 people. Key informant semi-structured interviews were conducted with traditional healers and the nurse of the health dispensary in Ahondo. In one hamlet (Amani Kouadiokro), no traditional healer was recognized by the whole population. Qualitative data were analyzed by the first author who participated in all interviews, made notes in writing from the tape-record version and extracted relevant information for the results presented here.

**Table 1:** Number of key informant interviews, focus group discussions, and questionnaires carried out in the five study localities of the Taabo health demographic surveillance system, south-central Côte d’Ivoire, in February 2012

	**Key informant interviews**		**Focus group discussions**				**Questionnaires**	
**Locality**	**Health staff**	**Traditional health practitioner**	**School-aged children**	**Women**	**Village authorities**	**Health staff**	**Children**	**Women**
Ahondo (village)	1	1	1	1	1	0	40	39
Amani-Kouadiokro (hamlet)	0	0	1	1	1	0	19	15
Katchénou (hamlet)	0	1	1	1	1	0	45	14
Sahoua (village)	0	1	1	1	1	0	13	22
Taabo Cité (town)	0	2	1	1	1	1	83	25
**Total**	**1**	**5**	**5**	**5**	**5**	**1**	**200**	**115**

### Statistical analysis

Questionnaire data were entered twice in Microsoft Access version 10.0 (2007 Microsoft Corporation). Double-entered datasets were compared using EpiInfo version 3.4.1 (Centers for Disease Control and Prevention; Atlanta, USA), and discrepancies were removed by going back to the original questionnaire. All data were analyzed using Stata version 10 (StataCorp.; College Station, USA).

Household socioeconomic status was calculated using an asset-based index [21]. Data on household assets (e.g., possession of a radio), housing characteristics (e.g., walls constructed with bricks), and the number of people per room were obtained from the existing Taabo HDSS database. Using principal component analysis (PCA) to weight the binary data of these variables, we subsequently divided the households into three socioeconomic groups (wealth tertiles): (i) very poor; (ii) poor; and (iii) least poor. Food was categorized according to indicators put forward by the World Health Organization (WHO) [22]. Responses from children and women are reported separately to easily visualize potential differences between the two age groups and because the administered questionnaires were slightly different. The mean prominence of reported variables (2, spontaneously reported; 1, probed; 0, not reported) was compared across study settings (town, village, hamlet) and between individuals who have been exposed to prior research and newly recruited participants, using the Kruskal-Wallis test and Wilcoxon rank-sum test, respectively. Significance was set as a p-value adjusted for ties <0.05.

## Results

### Socioeconomic characteristics of the study population

Table 2 shows that the socioeconomic status of participants differed among study setting. Whilst more than 60% of women and school-aged children residing in Taabo Cité belong to the least poor tertile, more than 90% of the participants living in hamlets belong to the poorest tertile of the population. Moreover, while 60.0% of the women and 95.2% of the children living in Taabo Cité attended school, the respective proportions were considerably lower in rural areas. In Taabo Cité, retailing is the main activity of 40.0% of women, whereas farming is the principal occupation of 86.2% of the women interviewed in hamlets. The villages of Ahondo and Sahoua represent an intermediate situation between town and hamlet for all parameters.

**Table 2:** Socioeconomic characteristics of the study population, stratified by setting and age group

**Characteristics**	**Town (N=108)**		**Village (N=114)**		**Hamlet (N=93)**	
	**Women**	**Children**	**Women**	**Children**	**Women**	**Children**
	***n *** **(%)**	***n *** **(%)**	***n *** **(%)**	***n *** **(%)**	***n *** **(%)**	***n *** **(%)**
**Sociodemographic indicator**						
Poorest	2 (8.0)	2 (2.4)	9 (14.8)	7 (13.0)	28 (96.6)	59 (90.8)
Poor	7 (28.0)	30 (36.1)	30 (49.2)	27 (50.0)	1 (3.5)	6 (9.2)
Least poor	16 (64.0)	51 (61.5)	22 (36.1)	20 (37.0)	0	0
**Mean age (years)**	20.0	9.0	21.6	8.9	22.2	8.8
**Female**	25 (100.0)	38 (45.8)	61 (100.0)	19 (35.9)	29 (100.0)	31 (48.4)
**Principal language of the interview**						
French	21 (84.0)	66 (79.5)	28 (45.9)	14 (26.4)	4 (13.8)	3 (4.7)
Other	4 (16.0)	17 (20.5)	33 (54.1)	39 (73.6)	25 (86.2)	58 (95.3)
**Went to school**	15 (60.0)	79 (95.2)	21 (34.4)	43 (81.1)	6 (20.7)	49 (76.6)
**Can read and write**	12 (48.0)	73 (87.6)	15 (24.6)	32 (60.4)	3 (10.3)	26 (40.6)
**Occupation**						
Farmer	1 (4.0)	N/A	33 (54.1)	N/A	25 (86.2)	N/A
Merchant	10 (40.0)	N/A	18 (29.5)	N/A	1 (3.5)	N/A
Housekeeper	6 (24.0)	N/A	8 (13.1)	N/A	1 (3.5)	N/A
Student	4 (16.0)	N/A	2 (3.3)	N/A	1 (3.5)	N/A
Other	4 (16.0)	N/A	0	N/A	1 (3.5)	N/A

N/A, not applicable.

### Local terms and representations of blood and anemia-related illnesses

Local terms reported by community chiefs for blood and anemia-related illnesses, and their approximate English translation, are summarized in Table 3. Whilst the word “blood” did exist in each local language with no difference in meaning, there was no direct translation for “anemia”. The local terms used to describe anemia-related illnesses indicate how these conditions are perceived by the population.

**Table 3:** Local terms for blood and anemia and their approximate translation into English in south-central Côte d’Ivoire

**Ethnic groups**	**Language groups**	**Local terms for blood**	**Local terms for anemia**	**Conceptual translation of local terms for anemia**
**Akan**	Abidji	*mbouo*	*mbouo ohou*	Blood is over
	Agni	*modja*	*modja wa wié*	Blood is over
	Akyé	*vûn*	*opou vûn*	Blood is over
			*o vûn ésè*	Blood decreased
	Alladjan	*inkrè*	*inkrè tro*	Blood is over
	Baoulé	*modja*	*modja wa vié*	Blood is over
			*modja wa kpêssou*	Blood decreased
			*modja djouman*	There is insufficient blood
**Krou**	Bété	*drou*	*drou yé bia*	Blood is over
**Gur (voltaic)**	Koulango	*tôm*	*tôm bayô*	Blood is lacking
			*tôm la*	Blood is over
	Senoufo	*chichan*	*chichan N’kwô*	Blood is over
			*chichan yôrôgo*	Blood decreased
	Tagwana	*dissiant*	*dissiant wo manni*	Blood is lacking
**North Mandé**	Malinké	*bassi; djoli*	*bassi banan*	Blood is over
			*bassi dôgô yala*	Blood decreased
**South Mandé**	Yacouba	*gno; abèr*	*gnon gnin,*	Blood is over
			*abèr yagnin*	Blood is over
	Gouro	*gnin*	*è gnan tara*	Blood is over

A representation is the constructed image, the meaning or the association people make with another element or condition. Table 4 summarizes the representations of blood and anemia-related illnesses among women and school-aged children. The representations of blood included four main categories: “something which is in the body”, “life”, “strength”, and “health”. Data from the FGDs elaborate these concepts:

“Blood makes the body work. Man is born with blood. If you don’t have blood, this means you are not a man. Blood is like the motor of man” (community chiefs in Taabo Cité).

**Table 4:** Local representations of blood and anemia among women and children, across study settings

**Characteristics**	**Town (N=108)**		**Village (N=114)**		**Hamlet (N=93)**	
	**Women**	**Children**	**Women**	**Children**	**Women**	**Children**
	***n *** **(%)**	***n *** **(%)**	***n *** **(%)**	***n *** **(%)**	***n *** **(%)**	***n *** **(%)**
**Representation of blood**						
In the body	7 (28.0)	47 (56.6)	6 (9.8)	28 (52.8)	4 (13.8)	19 (29.7)
Life	15 (60.0)	4 (4.8)	20 (32.8)	2 (3.8)	8 (27.6)	4 (6.3)
Health	1 (4.0)	1 (1.2)	4 (6.6)	2 (3.8)	4 (13.8)	6 (9.4)
Strength	0	1 (1.2)	22 (36.1)	1 (2.3)	9 (31.0)	11 (18.3)
Other	4 (16.0)	26 (31.3)	11 (18.0)	14 (26.4)	7 (24.1)	14 (21.9)
Do not know	0	4 (4.8)	1 (1.6)	7 (13.2)	0	16 (25.0)
**Representation of anemia-related illnesses**						
Death	14 (56.0)	58 (69.9)	21 (34.4)	25 (47.2)	10 (34.5)	43 (67.2)
Illness	10 (40.0)	15 (18.1)	37 (60.7)	16 (30.2)	16 (55.2)	12 (18.8)
Weakness	1 (4.0)	1 (1.2)	1 (1.6)	1 (1.9)	1 (3.5)	2 (3.1)
Other	0	5 (6.7)	5 (8.2)	4 (9.1)	1 (3.5)	4 (6.7)
Do not know	0	9 (10.8)	0	9 (17.0)	1 (3.5)	4 (6.3)

Spontaneous responses are reported. Multiple responses were permitted.

The 25% of children who suggest other ideas about blood typically explain, “blood is red water” or “blood is *bissap*” (a locally produced ice tea from hibiscus calyx). Anemia-related illnesses were mainly associated with either “death” or “illness”. Other representations of these conditions suggest that “this is bad stuff”, and “you cannot walk, you cannot do anything”.

### Relationship between anemia-related illnesses and local health problems

**Figure 1:**
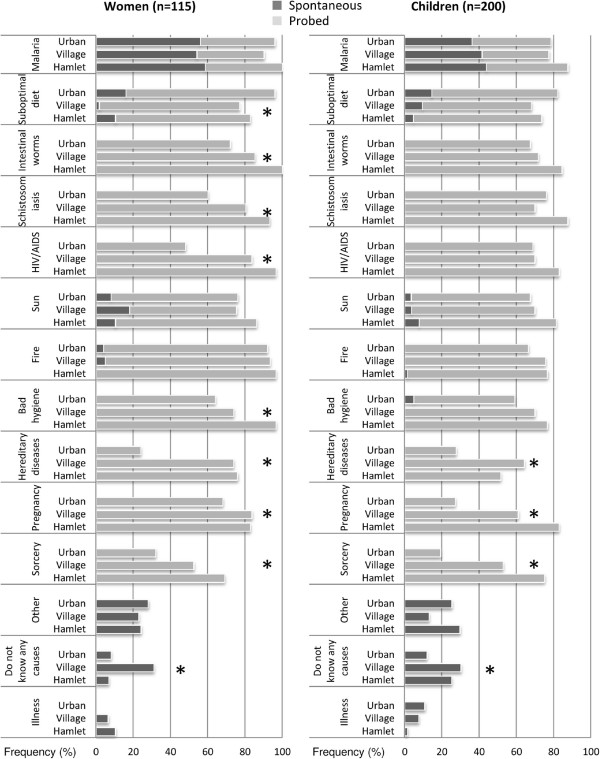
**Perceived causes of anemia among children and young women in south-central Côte d’Ivoire.** Spontaneous (dark grey) and probed (light grey) answers are reported and the mean prominence is compared across study settings with the Kruskal-Wallis test. Asterisks indicate p-value <0.05.

Figure 1 shows that participants identify various causes of anemia. Spontaneously, *djékouadjo* (malaria-like illnesses) is the cause reported most often, both by school-aged children and women. However, FGDs showed that people’s ideas about malaria differ from biomedical definitions of health workers:

“*Djékouadjo* might be caused by the sun, by hard work, by mosquitoes, or by the consumption of red oil and oily food” (women in Katchénou).

Indeed, sun, hard work, oily food, and mosquitoes were the most frequently reported causes of *djékouadjo*. Key informant interviews revealed that *djékouadjo* is a term which might group together different illnesses: the symptoms of *djékouadjo yassoua* (*yassoua* = male) resemble to severe or cerebral malaria, whereas *djékouadjo bla* (*bla* = female) refers to less severe febrile illnesses, and “*djékouadjo-ôklouè*” (*ôklouè* = yellow) is a febrile illness associated to yellow-colored eyes. Furthermore, there are cultural beliefs about how malaria-like illnesses cause anemia. In Amani Kouadiokro, for example, a woman explained that:

“The mosquito sucks your blood, and finishes it up, step by step”.

Diet, ill-health, fire, and sun are other important causes of anemia reported by both population groups. FGDs confirmed the results obtained from our questionnaire survey, as expressed by a woman in Amani Kouadiokro:

“Diseases like *djékouadjo*, from mosquitoes and flies, hard work, too many childbirths, or sitting too often next to the fire: these are all circumstances which can finish your blood”.

Fire was overall identified as an important cause of anemia and a traditional healer from Taabo Cité explained this causal relationship:

“When you are sitting next to the fire, the fire draws your blood”.

Other more technical considerations argue that:

“You see it when you put an animal on a fire. Blood becomes solid, coagulates and can no more circulate within the body” (communities’ chiefs in Taabo Cité).

Whilst women from rural areas more often reported schistosomiasis, intestinal worms, HIV/AIDS, and lack of hygiene as causes of anemia, the mean prominence of suboptimal diet was significantly higher in Taabo Cité. FGDs with village authorities revealed that a couple of food plants are indeed seasonal (e.g., mangos, yams, and eggplants). However, according to village authorities, the majority of food is available throughout the year and unvaried diet is due to limited food preference and/or availability characteristic of each ethnic group. Furthermore, both children and women from rural areas reported more often that sorcery might trigger anemia, compared with children and women living in town. A traditional healer in Taabo Cité explained:

“Sorcerers might draw your blood”.

Communities’ chiefs detailed this process as follows:

“There are two types of blood: the sweet one, which has not been prepared, and the sour one, which has been prepared with traditional medicine. Sorcerers only drink unprepared, sweet blood.”

Another recurrent cause of anemia which emerged during FGDs was *coco* (hemorrhoids):

“*Coco* is not a good thing. It spoils your blood and then you get very tired” (traditional healer in Katchénou).

The questionnaire survey showed that one-fifth of children and women were not able to spontaneously quote any cause of anemia.

**Figure 2:**
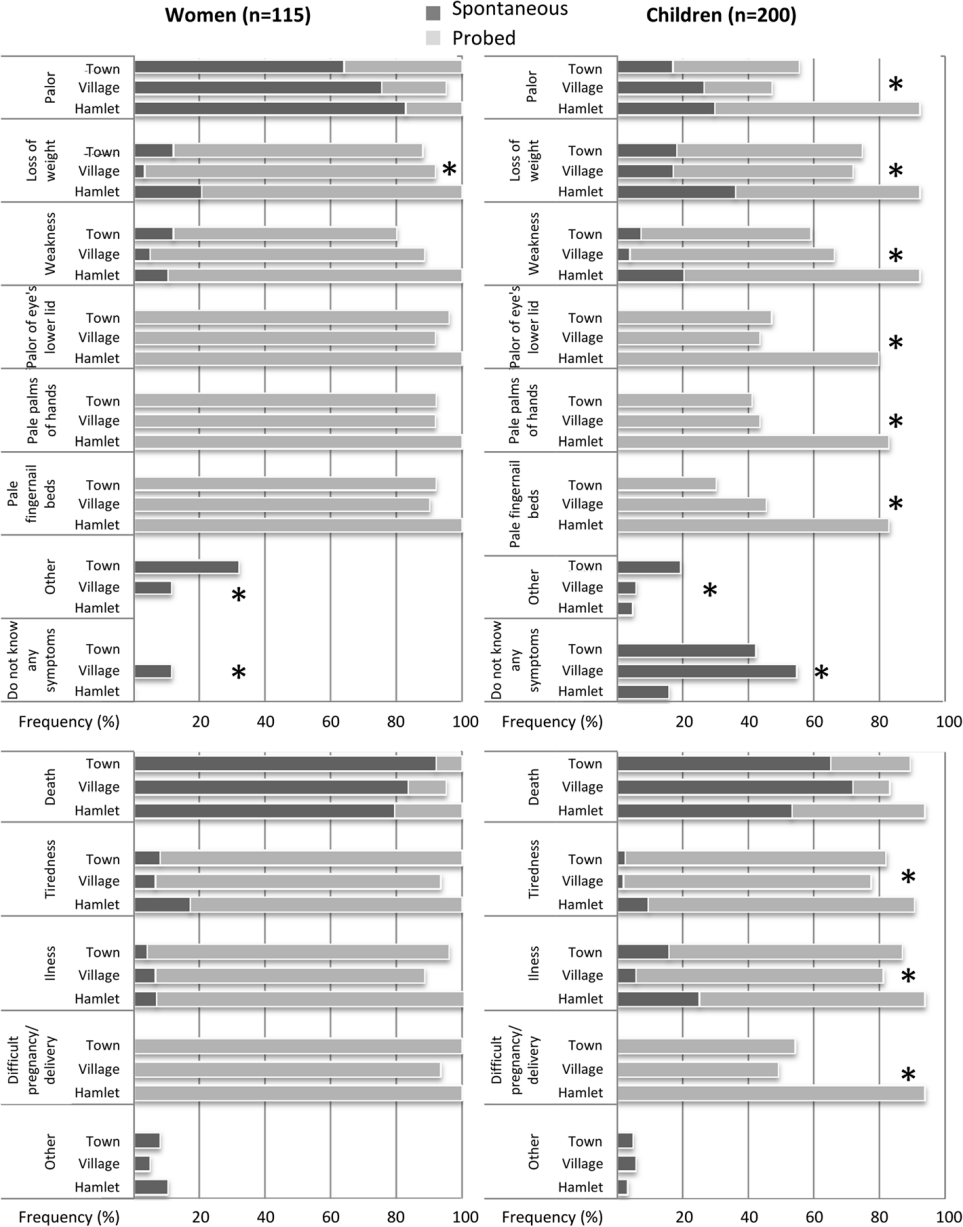
**Perceived symptoms and consequences of anemia among children and young women in south-central Côte d’Ivoire.** Spontaneous (dark grey) and probed (light grey) answers are reported and the mean prominence is compared between study settings with the Kruskal-Wallis test. Asterisks indicate p-value <0.05.

Participants reported three main types of symptoms associated with anemia. They include pallor, loss of weight, and weakness/tiredness (Figure 2). The qualitative data from the FGDs clarify these findings:

“Your body is white, your eyes are white, when you eat something, you vomit it; you lose weight, you get dizzy, you are weak and your body heats up” (women in Katchénou).

Of note, mean prominence of pallor as a symptom of anemia was significantly lower among children and women who had been exposed to prior research than among newly recruited participants. Three main consequences of anemia were identified among spontaneous answers from women and school-aged children: weakness, illness, and death. Death is the most frequently reported consequence of anemia, both by women (84.4%) and children (63.0%). Four children stated that “anemia is happiness”, “anemia gives strength”, and “anemia gives health”.

The two main sources of knowledge about anemia in both population groups stem from other family members and from medical staff at health centers (data not shown). The school was mentioned as another source of knowledge, whereas no participants spontaneously reported television or radio as their source of information.

### Help-seeking and risk-related reported behaviors for anemia-related illnesses

Whilst women mainly reported the consumption of leafy vegetables as an effective measure to prevent anemia (Table 5), school-aged children stated that good food, meat and fish, and *bissap* are effective to prevent anemia. Both women and school-aged children spontaneously declared that visits to the health center in case of ill-health or during health check-ups are important to prevent anemia. FGDs with village authorities and women emphasized that medicine and tonics are important preventive measures against anemia:

“If you don’t want to have anemia, you have to take medicine. There is medicine you get from the doctor but we also have leaves, here, which give you blood” (communities’ chiefs in Taabo Cité).

**Table 5:** Reported behaviors toward preventive measures against anemia in the Taabo HDSS, south-central Côte d’Ivoire

**Characteristics**	**Women (N=115)**				**Children (N=200)**			
	**Spontaneous *n *** **(%)**	**Probed *n *** **(%)**	***p *** **(KW)**	***p *** **(WRS)**	**Spontaneous *n *** **(%)**	**Probed *n *** **(%)**	***p *** **(KW)**	***p *** **(WRS)**
Varied diet^a^	26 (22.6)	82 (71.3)	0.122	0.206	46 (23.0)	121 (60.5)	0.402	0.048
Protection from malaria^b^	15 (13.0)	90 (78.3)	0.053	0.395	15 (7.5)	158 (79.0)	0.110	0.899
Drink *bissap*	14 (12.2)	92 (80.0)	0.073	0.689	36 (18.0)	129 (64.5)	0.083	0.553
Eat meat, fish	16 (13.9)	76 (66.1)	0.046	0.191	10 (20.0)	132 (66.0)	0.448	0.058
Traditional medicine	4 (3.5)	95 (82.6)	0.417	0.641	2 (1.0)	143 (71.5)	0.005	0.267
Prayers, spiritual practices	0	65 (56.5)	0.005	0.014	5 (2.5)	124 (62.0)	<0.001	0.131
Good hygiene	3 (4.5)	0	0.443	0.707	13 (6.5)	N/A	0.075	0.983
Eat leafy vegetables	29 (25.2)	N/A	0.705	0.618	5 (2.5)	N/A	0.694	0.157
Drink Coca-Cola	9 (7.8)	N/A	0.022	0.238	13 (6.5)	N/A	0.001	0.985
Eat tomatoes	11 (9.6)	N/A	0.028	0.109	10 (5.0)	148 (74.0)	0.679	0.146
Visit health centers^c^	32 (27.8)	76 (66.1)	0.106	0.433	34 (17.0)	132 (66.0)	0.110	0.794

Mean prominence of reported variables (coded 2 if spontaneously reported; 1, if reported after probing; and 0 if not reported) of women and children are reported. Mean prominence of reported variables have been compared across study settings and between people who had been exposed to prior research and those newly recruited with the Kruskal-Wallis (KW) and Wilcoxon rank-sum (WRS) tests, respectively, and associated p-values are reported. Multiple responses were permitted.

^a^ Spontaneous responses include “we shall eat enough/good food/vitamins”, “we shall watch for our alimentation” (see Discussion).

^b^ Three types of spontaneous answers are grouped here: “not walking under the sun”, “protection against *djékouadjo*”, and “protection against mosquitoes”.

^c^ People either mention “we shall do regular health check-up” or “we shall go to the doctor when we are ill”.

N/A, not applicable.

Other behaviors reported by more than 5% of the participants include consumption of meat, Coca-Cola, and tomatoes, as well as protective behaviors against *djékouadjo.* Prayers and spiritual practices were more often reported by individuals from rural areas, whilst drinking Coca-Cola was only reported by inhabitants of Taabo Cité. During FGDs, several children reported they should be clean and behave well to prevent anemia-related illnesses:

“You should neither play in rubbish nor in filth. You should not contradict, nor bite people” (children from Katchénou).

Most of the women interviewed told us they would go to the health center if they were anemic, whilst 17.3% would take traditional medicine and 6.4% put forward other curative measures (data not shown).

Table 6 shows that slightly more people are sleeping under long-lasting insecticidal nets (LLINs) in Taabo Cité than in villages and hamlets, although the difference lacked statistical significance (*χ*^2^=3.99, p=0.136). More than half of the school-aged children and women reported having eaten meat or fish the day before the interview. Twenty-four-hour food recall indicated that most participants did not consume food from three or more groups in a single meal on the day preceding the interview.

**Table 6:** Preventive and help-seeking behaviors, experience of illness and treatment use in relation to anemia in south-central Côte d’Ivoire

**Characteristics**	**Town (N=108)**		**Village (N=114)**		**Hamlet (N=93)**	
	**Women**	**Children**	**Women**	**Children**	**Women**	**Children**
	***n *** **(%)**	***n *** **(%)**	***n *** **(%)**	***n *** **(%)**	***n *** **(%)**	***n *** **(%)**
Proportion of people sleeping under LLINs^a^	0.51		0.45		0.37	
Ate food containing heme-iron^b^	18 (72.0)	38 (50.7)	38 (65.5)	24 (54.6)	26 (89.7)	51 (85.0)
Food from ≥3 groups in a single meal^b^	10 (40.0)	19 (25.7)	20 (34.5)	12 (27.9)	10 (34.5)	26 (43.3)
Experienced anemia	8 (32.0)	10 (13.3)	10 (17.2)	2 (4.6)	6 (20.7)	3 (5.0)
Anemia diagnosed in a health center	7 (87.6)	6 (60.0)	9 (90.0)	2 (100.0)	2 (33.3)	1 (33.3)
Received modern medicine	8 (100.0)	10 (100.0)	10 (100.0)	2 (100.0)	6 (100.0)	3 (100.0)
Received medicine from a traditional healer	1 (12.5)	2 (20.0)	3 (30.0)	0	3 (50.0)	3 (100.0)

^a^ The estimated number of individuals who slept under a LLIN the night preceding the interview was obtained from the Taabo HDSS database (October 2011). Hence, they are not specific to children and women but refer to the whole population of the localities.

^b^ 24-hour recall.

Several participants – mainly young women – acknowledged that they have had anemia. The mean prominence of having experienced anemia was neither significantly different across study settings, nor between individuals who have been exposed to prior research and newly recruited participants. All participants who experienced anemia reported that they received modern medicine to treat this condition. However, not all cases of anemia were diagnosed and treated in health centers. In addition to modern treatments, some participants received medicine from traditional healers, mainly in the hamlets (100% of women and 50% of children). Two women and one child knew they had received iron supplements and two women identified other tonics. Other children and women were unable to identify which kind of medicine they received from the health staff.

## Discussion

To our knowledge, this is the first study to investigate local concepts of blood and various anemia-related illnesses among school-aged children and young women, and their potential public health implications for risk-related and help-seeking behaviors, in a multiethnic setting of West Africa. Using a mixed methods approach to examine quantitative and qualitative data, we found that although the biomedical term anemia does not exist in the main local languages, the semantic form of the ancient Greek term *ἀναιμία*, meaning without blood, corresponds to the concepts used in local folk languages to report this condition. Our results reveal biomedical and sociocultural features of the knowledge of children and women for anemia-related illnesses. These representations appear to be connected to the risk-related and help-seeking behaviors reported by children and women and to differ from professionally recognized causes of anemia.

### Limitations

Our study has several limitations. First, although we identified different terms related to mild and severe anemia during the initial meeting with traditional authorities, no distinction was made between these two categories in our questionnaire survey. Such a distinction would have required very subtle terminology, particularly for school-aged children, but this was not feasible given our tight time schedule and limited human and financial resources to conduct the study. Future KAPB surveys pertaining to anemia might investigate the implications of “relative” and “absolute” terms in relation to people’s behaviors and practices. In turn, such knowledge might provide important information for locally adapted communication strategies to prevent anemia.

Second, our study lacks direct observational components, which might have given more credibility and weight to the findings from the questionnaire survey. Indeed, previous studies have shown that reported and observed results differ quite considerably [23]. Nevertheless, such observations would have been challenging in the case of anemia, since cases are difficult to identify without clinical examinations. We suggest that future similar studies, particularly hospital-based surveys, might integrate an observational component.

### Representations of blood and anemia-related illnesses

The difference in representations of blood across study settings might be associated with the main activity of the population that differs in rural and urban areas. Whilst inhabitants of villages and hamlets are mainly engaged in subsistence farming, a considerable number of individuals living in Taabo Cité are occupied in the tertiary sector (e.g., merchants and staff of the hospital, the school and management of the Taabo dam), or are attending high school. This observation might explain why people’s conception of blood mainly refers to strength in the more rural areas, where this condition is crucial for being productive in daily agricultural activities. People use semantic constructions associated to the ill-health aspects of anemia. These constructions are related to relative and absolute concepts of anemia-related illnesses. Relative concepts include “blood decreased” and “blood is not enough” and these descriptions relate to non-severe forms of anemia. This concept of anemia-related illnesses is found in other communities of West Africa and throughout the world [13,15]. “Blood is over” indicates the absolute concept of anemia. Hence, anemia is also considered as a severe illness and, indeed, a cause of death.

The important differences we observed throughout the interview between spontaneously reported and probed answers might be explained by different levels of importance in the association that people construct between etiological agents and anemia. The large number of individuals who spontaneously reported *djékouadjo* (malaria-like illnesses) as a cause of anemia suggests that, in the Taabo HDSS, people construct a strong relationship between anemia and malaria-like illnesses. This association might be explained by the severity of some malarial anemia, which forces people to seek care at the hospital. As people do not frequently visit health centers, the information they receive there might stick into their mind. In contrast, illnesses like schistosomiasis were not spontaneously reported, most likely because there are no obvious causes of anemia for the population. However, probing schistosomiasis as a potential cause of anemia showed that most people think this relation exists. This observation seems quite obvious by the local terminology of schistosomiasis, which refers to “the one who urinates blood”. A similar explanation can be given for HIV/AIDS; although people do not spontaneously think about it as a cause of anemia, they considerate AIDS as a blood-related disease which renders people weak. Hence, upon probing, people agree that AIDS can cause anemia. Another factor which might explain the discrepancy between spontaneously and probed answers is, particularly among children, that they are shy or unable to give their own opinion. However, the very low proportion of participants who positively answered to absurd questions such as “Is health a consequence of anemia?” indicates that most respondents understood the questions and gave meaningful answers.

Health centers and other family members were identified as the main sources of knowledge about anemia, which corresponds to the sources of knowledge identified in a previous study about the use of LLINs in Côte d’Ivoire [24]. However, it is worth mentioning that health facilities are almost exclusively used as curative health structures. This may limit effective communication between the community and the health system for effective preventive medicine. Considering that, in the current study area, a household consists, on average, of eight individuals, it is not surprising that within-family communication was identified as an important source of information. Television and radio are less important sources of knowledge, inasmuch as the infrastructure has little or no support for this form of media. At the time of our study, Katchénou and Amani Kouadiokro were still not connected to the power grid. However, a few households owned a generator, which was used for various purposes, including watching television.

### Relationship between anemia-related illnesses and local health problems

Our results revealed that the knowledge of participants about various anemia-related illnesses was based either on biomedical or sociocultural concepts and a clear distinction was often blurred. The biomedical dimension includes biomedical causes and preventive attitudes and reported behaviors against anemia, as shown in previous studies [4,5,9]. The sociocultural dimension groups together beliefs, attitudes, and reported behaviors, which require further in-depth investigations.

Biomedical causes include pregnancy, *djékouadjo* (malaria-like illnesses) and suboptimal diet, which is in line with our prior research in the Taabo HDSS [9,10] and that of others elsewhere in sub-Saharan Africa [5,25]. However, FGDs revealed that *djékouadjo* is not a synonym of malaria. Such local cultural distinctions between several malaria-like illnesses were identified elsewhere in Africa and turned out to be important parameters to take into account when developing a prevention program [26]. The perceptions of food and nutrition are also complex. People talk about good food or large quantity of food rather than iron-rich food or a diversified diet, emphasizing the divergence within the so-called “biomedical” causes as understood by the health staff and by the population.

Sociocultural causes of anemia-related illnesses include the sun, fire, and sorcerers. The relation between sun or fire and anemia mainly relates to the effect of heat; whilst sun causes anemia through sweating, fire impacts on the fluidity of blood. Interestingly, indoor biofuel smoke has been identified as a risk factor for anemia [27], and the effect of outdoor biofuel cooking on Hb levels may be worth investigating. The belief that sorcerers can cause anemia, mainly found among rural communities in the present studies, is encountered in several communities about different diseases, including malaria-like illnesses and tuberculosis [17,28].

Color was an important parameter in the diagnosis of anemia, particularly for people living in the most rural areas. The representations of white and yellow colors as signs of ill-health are also found in the study of tuberculosis, where white cough and white body are signs of disease [29]. Although the loss, rather than the gain, of weight was associated with anemia, people also mentioned that anemic individuals might become bigger, specifying that “this is not good fat”, referring to swelling and edemas.

Both children and women identified three main consequences resulting from anemia-related illnesses: illnesses, tiredness, and death. On the one hand, most interviewees spontaneously reported death as the ultimate consequence of anemia-related illnesses. On the other hand, tiredness and illness both impact on working capacity, productivity, and financial resources, which is particularly important in subsistence farming communities, as observed here for the Taabo HDSS.

### Help-seeking and risk-related attitudes and behaviors for anemia-related illnesses

Knowledge and beliefs of children and women about various anemia-related illnesses affect their attitudes and behaviors toward preventive measures against anemia. Although *djékouadjo* was identified as an important cause of anemia, few people mentioned the use of LLINs as an effective preventive measure against anemia. These considerations explain why few people think about sleeping under a LLIN as a preventive measure against anemia and confirm previous observations from Côte d’Ivoire, which showed that although 73% of interviewees utilized nets to prevent nuisance from mosquitoes, only 9% thought this measure may protect them from malaria [30]. According to the Taabo HDSS database, almost half of the people are now sleeping under a net, with a slightly higher coverage in Taabo Cité (the only small town) than in rural areas, which is much higher than in mid-2008 when the Taabo HDSS was established [31,32]. The considerable increase of LLIN coverage can be explained by a recent national distribution campaign carried out between November 2010 and July 2011 and confirms that people use LLINs without being aware of their preventive effect against *djékouadjo*. Similar sociocultural concepts about malaria-like illnesses have been reported from other communities across the world, whose local denominations do not exactly correspond to the biomedical term malaria [12,26,33-37].

Children and women consider food as an important issue in the prevention of anemia. However, the recurrent expressions “eat well” and “good food” had different meanings, depending on the population group interviewed. Whilst the consumption of leafy vegetables and vitamins are sometimes reported by adults, it is more the quantity than the quality that matters to children, although the final goal is the same for all age groups: to gain strength. Beside leafy vegetables and meat, children and women reported other foodstuffs and drinks they may use to prevent anemia: *bissap* and tomatoes were more reported in rural areas whilst Coca-Cola was exclusively quoted by people living in Taabo Cité. These fluids were also reported by traditional healers. Interestingly, the aforementioned foodstuffs and drinks, as well as local herbal teas used to prevent or treat anemia, are all dark red-colored. Such a relationship between red-colored drink and foodstuffs with anemia has been reported for other communities [38]. Our findings therefore suggest that people may build a relationship between color and anemia, not only for diagnostic purposes, but also for curative measures. *Bissap* may be worth further investigating as other groups of researchers reported a high content in vitamin C, one component that can improve iron absorption [39].

Most of the women interviewed (84.4%) said they would seek care at a health center if they suffer from anemia and 69.2% of the respondents who experienced anemia said they got their diagnosis from a health center. However, health workers consistently reported that people visit health centers at a late stage of disease. In case of ill-health, people usually visit traditional healers first. FGDs corroborate these findings; in case traditional medicines do not improve the subject’s health status, then he/she may visit a health center. These observations suggest that different concepts of anemia-related illnesses (relative *versus* absolute) may be associated with different behaviors. However, future studies should seek a clearer distinction between these concepts to investigate whether they are associated to specific behaviors and might therefore influence public health actions.

### Public health implications of local cultural concepts and ideas about anemia

The discrepancy between professionally recognized causes of anemia and accompanying preventive and curative measures, and local culturally reported causes and ideas about anemia-related illnesses have important ramifications for public health. Indeed, our results indicate that there are additional local culturally identified causes of anemia (e.g., sun, fire, and sorcerers), and that so-called biomedical factors are understood differently, which influence the prevention and control of anemia by the population and the health staff.

Whilst the health staff put emphasis on the quality, rather than the quantity of food for preventing anemia, we noted a different pattern within the population. Less than 40% of the participants effectively had a balanced diet on the day before the interview. Most people reported that they have eaten meat or fish on the day preceding the interview, but this response would need to be confirmed by active food records. Indeed, in our prior epidemiological study, we found high prevalence of iron, vitamin A (in infants and children), and riboflavin deficiencies [9], which indicate that the local diet does not fulfill people’s micronutrients requirements. Moreover, the considerable prevalence of inflammation certainly contributes to the local burden of malnutrition through preventing efficient micronutrients absorption [9,10]. The situation of the Taabo HDSS, located in the V-Baoulé where the rain forest meets the Savannah area offers many agricultural and fishing opportunities. However, our results highlight the influence of geographical origins on diet customs. Whilst food of communities from the North (e.g., Malinké and Sénoufo) is based on cereals (i.e., maize, sorghum, millet, and rice), tubers and plantain are the main staple food of ethnic groups from the South [40,41].

The potential public health implications of various types of *djékouadjo* are as follows. Considering that not all *djékouadjo* are thought to be transmitted by mosquitoes, this affects people’s behavior, inasmuch they do not associate LLINs to malaria prevention and referral to the health system is only done for the most severe cases of malaria and anemia. Of note, as blood transfusions are not available in Taabo General Hospital, severe anemia cases are referred to other larger hospitals further away. This delay in seeking prompt and effective care and the consequence on population health has been studied in different communities throughout the world [13,42-45]. In the case of malaria, which is responsible for most cases of severe anemia in sub-Saharan Africa, low socioeconomic status, distance to the nearest health center, cost of care, and perceived adverse events of modern medicine are among the key issues people put forward not to seek prompt care at health facilities [13,42,43]. The consequences include a reduced efficacy of treatment, and hence, a higher probability of complications and even death. Data from health registries at Taabo General Hospital and Ahondo health center indicate that during the year 2011, 64/5,539 (1.2%) and 33/1,138 (2.9%) consultations, respectively, were diagnosed with severe anemia. Mild and moderate cases of anemia were not systematically registered, indicating that the consequences of chronic, non-severe anemia might be underestimated by the health staff.

## Conclusions

Our study identified different local concepts of anemia, specifying two levels of severity. Children and women construct relations between these concepts and health problems. Although malaria, nutrition, and hygiene are identified as important issues affecting the quantity of blood, they do not refer to similar concepts within the health staff and the population. These findings are of public health relevance, since people’s cultural ideas about anemia are related to their risk-related and help-seeking behaviors and might, in turn, affect their health status. These findings will have to be considered when developing health programs, inasmuch a misunderstanding of biomedical terms between the health system and the community might undermine any intervention. Although some differences were found between the three study settings, the overall concept, knowledge, and behaviors related to anemia were similar, and hence a uniform strategy may be used to develop education and intervention programs to reduce the prevalence of anemia in the Taabo HDSS and perhaps elsewhere in Côte d’Ivoire. Coupled to health and nutritional education, school canteens should be considered as 80% of children aged 8–10 years attend school. In addition, increasing the coverage of LLINs, accompanying by a better understanding of why sleeping under a LLIN is important, and health system strengthening must be further improved. Household-based education campaigns should be explored as an entry point to decrease the burden of anemia in sub-Saharan Africa.

## Competing interests

The authors declare no competing interests.

## Authors’ contribution

MKDK, AAR, NNA, RW, EKN, and JU, participated to the conception and design of the study; MKDK and AAR conducted the study and analyzed the data; MKDK, AAR, MGW, EKN, and JU interpreted the results; MKDK, AAR, NNA, RW, MGW, EKN, and JU have been involved in drafting the manuscript or revising it critically for important intellectual content; MKDK, AAR, NNA, RW, MGW, EKN, and JU have given final approval for the final version of the paper to be published; all authors read and approved the final manuscript.

## Pre-publication history

The pre-publication history for this paper can be accessed here:

http://www.biomedcentral.com/2052-1839/13/5/prepub
